# Durable response to vebreltinib in heavily pretreated *MET*-amplified intrahepatic cholangiocarcinoma: a case report

**DOI:** 10.1093/oncolo/oyag409

**Published:** 2026-10-13

**Authors:** Yingying Hang, Lu Bai, Hsiquan Hsu, Ying Chen, Fufeng Wang, Wuyan Xu, Xia Zhen, Zihao Wang, Yuan Li, Jun Qian

**Affiliations:** Department of Oncology, Affiliated Hospital of Nanjing University of Chinese Medicine, Jiangsu Provincial Hospital of Chinese Medicine, Nanjing 210029, China; The First Clinical Medical College, Nanjing University of Chinese Medicine, Nanjing 210023, China; Department of Oncology, Affiliated Hospital of Nanjing University of Chinese Medicine, Jiangsu Provincial Hospital of Chinese Medicine, Nanjing 210029, China; Jiangsu Collaborative Innovation Center of TCM Cancer Prevention and Treatment, Nanjing 210023, China; Department of Oncology, Affiliated Hospital of Nanjing University of Chinese Medicine, Jiangsu Provincial Hospital of Chinese Medicine, Nanjing 210029, China; The First Clinical Medical College, Nanjing University of Chinese Medicine, Nanjing 210023, China; Department of Oncology, Affiliated Hospital of Nanjing University of Chinese Medicine, Jiangsu Provincial Hospital of Chinese Medicine, Nanjing 210029, China; Jiangsu Collaborative Innovation Center of TCM Cancer Prevention and Treatment, Nanjing 210023, China; Geneseeq Research Institute, Nanjing Geneseeq Technology Inc, Nanjing 210032, China; Geneseeq Research Institute, Nanjing Geneseeq Technology Inc, Nanjing 210032, China; Department of Oncology, Affiliated Hospital of Nanjing University of Chinese Medicine, Jiangsu Provincial Hospital of Chinese Medicine, Nanjing 210029, China; Jiangsu Collaborative Innovation Center of TCM Cancer Prevention and Treatment, Nanjing 210023, China; Department of Oncology, Affiliated Hospital of Nanjing University of Chinese Medicine, Jiangsu Provincial Hospital of Chinese Medicine, Nanjing 210029, China; Jiangsu Collaborative Innovation Center of TCM Cancer Prevention and Treatment, Nanjing 210023, China; Department of Pathology, Affiliated Hospital of Nanjing University of Chinese Medicine, Nanjing 210029, China; Department of Oncology, Affiliated Hospital of Nanjing University of Chinese Medicine, Jiangsu Provincial Hospital of Chinese Medicine, Nanjing 210029, China; The First Clinical Medical College, Nanjing University of Chinese Medicine, Nanjing 210023, China; Jiangsu Collaborative Innovation Center of TCM Cancer Prevention and Treatment, Nanjing 210023, China

**Keywords:** *MET* amplification, intrahepatic cholangiocarcinoma, vebreltinib, targeted therapy, case report

## Abstract

**Background:**

*MET* amplification, although detected in only 2%-5% of intrahepatic cholangiocarcinomas (ICC), represents an actionable oncogenic driver. Treatment options after failure of first-line immunochemotherapy remain limited, and clinical data on vebreltinib, a novel highly selective *MET* inhibitor, in this molecular subset are scarce.

**Case Presentation:**

A 61-year-old woman with metastatic ICC harboring high-level *MET* amplification (tissue gene copy number, 26.79) experienced disease progression on first-line durvalumab plus gemcitabine/cisplatin, second-line liposomal irinotecan/fluorouracil plus lenvatinib and durvalumab, radiotherapy, and transarterial chemoembolization. Following a brief period of vebreltinib (200 mg twice daily) plus durvalumab that was interrupted by grade 4 myelosuppression, the patient continued on vebreltinib monotherapy after hematologic recovery. Restaging computed tomography demonstrated a partial response (PR), with carbohydrate antigen 19-9 normalizing (a 99.2% reduction). This response lasted over 6 months until eventual disease progression.

**Conclusion:**

To our knowledge, this is the first report of vebreltinib monotherapy inducing a deep and durable partial response in heavily pretreated, *MET*-amplified ICC, with the response lasting over 6 months despite eventual disease progression. This case underscores the critical value of comprehensive molecular profiling after first-line treatment failure to identify actionable drivers such as *MET* amplification.

Key points

- Vebreltinib monotherapy induced a deep and durable partial response (carbohydrate antigen 19-9 reduction 99.2%) lasting over 6 months in a heavily pretreated patient with *MET*-amplified ICC, although eventual disease progression occurred.
- High-level *MET* amplification (tissue gene copy number 26.79) can confer exceptional sensitivity to *MET* inhibition in intrahepatic cholangiocarcinoma, even after multiple lines of prior therapy.
- This case underscores the critical importance of comprehensive molecular profiling after first-line treatment failure to identify actionable drivers such as *MET* amplification.

## Patient story

A 61-year-old Chinese woman first experienced epigastric pain in June 2025 but did not seek medical attention. The pain worsened in July 2025, prompting hospital consultation. Physical examination revealed right upper quadrant tenderness, and serum CA19-9 was 6147 U/mL. Magnetic resonance imaging demonstrated multiple liver lesions, the largest measuring 39 × 26 mm with rim enhancement, and enlarged porta hepatis and retroperitoneal lymph nodes (largest 18 × 11 mm). Positron emission tomography-CT showed multiple hypermetabolic lesions in the liver parenchyma and hypermetabolic lymph nodes in the porta hepatis and retroperitoneum, consistent with metastases. Biopsy of a liver lesion confirmed adenocarcinoma (Figure 1). Based on these findings, the patient was diagnosed with intrahepatic cholangiocarcinoma (ICC), stage IVB (cT2N1M1) (Figure 3A).

**Figure 1.**
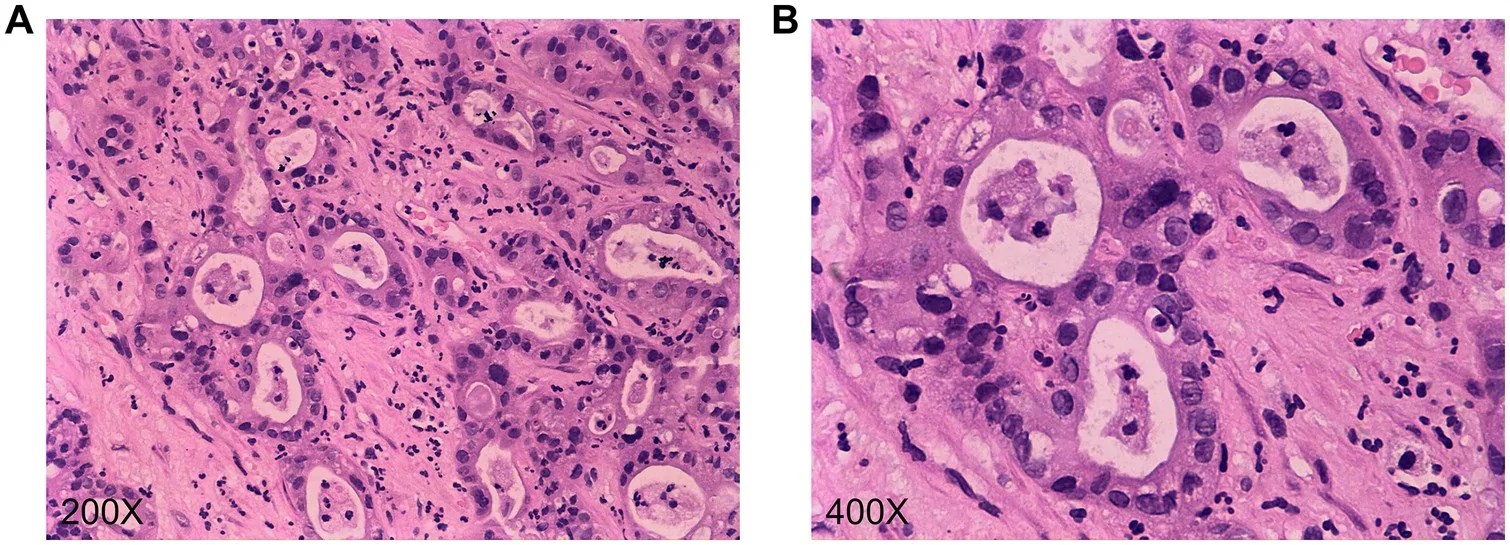
Histopathological findings from the liver core-biopsy specimen of intrahepatic cholangiocarcinoma. Hematoxylin-and-eosin (H&E) staining demonstrates poorly differentiated adenocarcinoma, characterized by pleomorphic malignant epithelial cells, high nuclear-to-cytoplasmic ratio, nuclear hyperchromasia, and abortive glandular architecture consistent with biliary-origin malignancy. (A) Low-power magnification, ×200; (B) high-power magnification, ×400.

She first received first-line therapy with durvalumab (1500 mg, day 1), gemcitabine (1.4 g, days 1 and 8), and cisplatin (40 mg, days 1 and 8) every 3 weeks for 3 cycles. She had stable disease as best response but progressed radiologically after approximately 3 months (Figure 3A). After multidisciplinary team (MDT) discussion, second-line therapy was initiated on October 16, 2025, consisting of liposomal irinotecan (75.32 mg), fluorouracil (0.5 g bolus plus 3.5 g continuous infusion over 46 h), and lenvatinib (8 mg once daily) every 2 weeks. On November 4, 2025, 1 cycle of durvalumab (1500 mg) was added. Because of slowly rising tumor markers, she received local radiotherapy (Bragg mode: PTV1 1 Gy, PTV3 3 Gy, PTV5 5 Gy) on December 3, 2025, concurrently with durvalumab and chemotherapy. For further local control, transarterial chemoembolization (TACE) with epirubicin (20 mg) and lipiodol was performed on December 22, 2025. After TACE, to mitigate myelosuppression, the fluorouracil continuous infusion dose was reduced from 3.5 to 3.0 g on December 26, 2025. Despite these treatments, a CT scan on January 14, 2026 showed disease progression: the largest liver lesion increased to 53 × 34 mm, and multiple enlarged retroperitoneal and iliac lymph nodes were noted; CA19-9 had risen to 3876 U/mL.

## Molecular tumor board

Targeted next-generation sequencing (NGS) of the liver biopsy specimen and matched plasma identified focal *MET* copy number amplification, with a corrected gene copy number (GCN) of 26.79 in tumor tissue and 9.65 in plasma ctDNA (Figure 2). A *TP53* exon 5 p. F134L missense mutation was also detected; the tumor was microsatellite stable with a low tumor mutational burden (TMB; 4.1 mut/Mb) (Supplementary Method). The key molecular findings from NGS are summarized in Table 1. The full NGS results are provided in Supplementary Dataset.

**Figure 2.**
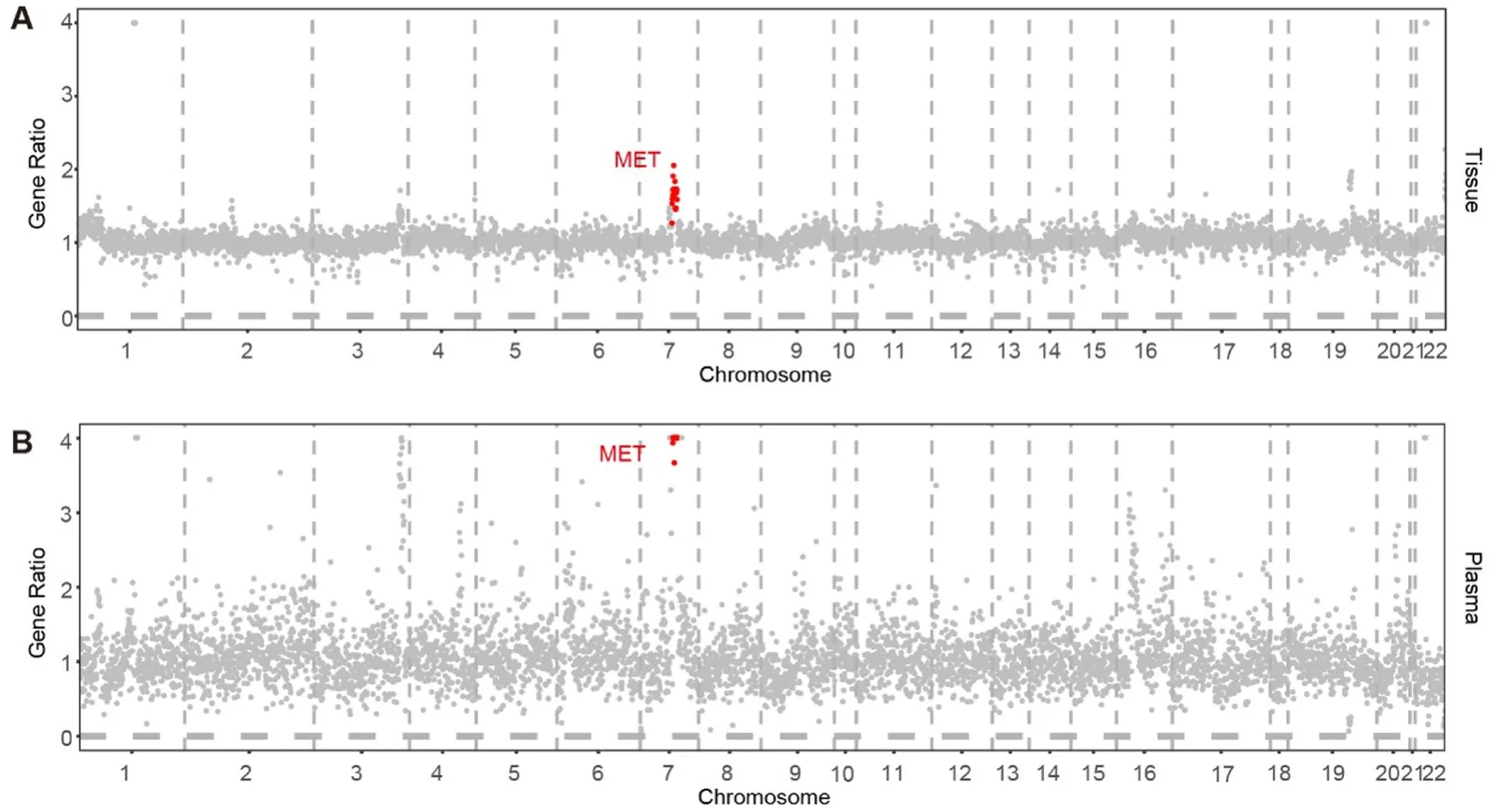
Molecular analysis of *MET* amplification. Copy number variation profiles demonstrated focal *MET* amplification on chromosome 7 in both tumor tissue and matched plasma. (A) Tumor tissue showed high-level focal *MET* amplification, with a corrected gene copy number (GCN) of 26.79. (B) Matched plasma ctDNA also showed focal *MET* amplification, with a corrected GCN of 9.65. The dots represent normalized gene-level copy ratios across chromosomes, with the peaks indicating the MET locus. Corrected GCN values were adjusted using an assay-specific algorithm and should not be directly inferred from raw copy ratios.

**Table 1.** Molecular findings from targeted next-generation sequencing of tumor tissue and matched plasma.

Parameter	Finding
**Sample type**	Liver biopsy tissue/matched plasma
**Detection method**	Targeted NGS
***MET* amplification**	Tissue: corrected GCN 26.79 Plasma ctDNA: corrected GCN 9.65
**Co-occurring mutation**	*TP53* exon 5 p. F134L missense
**Microsatellite status**	MSS
**Tumor mutational burden**	4.1 mut/Mb (low)

Abbreviations: GCN, gene copy number; MSS, microsatellite stable; NGS, next-generation sequencing; TMB, tumor mutational burden.

Based on the NGS-detected *MET* amplification, the case was discussed at the institutional multidisciplinary tumor board, and the MDT recommended initiating *MET* inhibitor therapy. Given that no *MET* inhibitor is formally approved for ICC, the treatment decision was based on the potent antitumor activity of vebreltinib demonstrated in clinical trials, its clinical accessibility through a compassionate use program in China, its favorable pharmacokinetic profile, and the patient’s preference after thorough discussion. After full discussion with the patient and her family, vebreltinib (200 mg twice daily) plus durvalumab (1500 mg) was started on January 16, 2026, through a compassionate use program in China. Three days later (January 19, 2026), she received an additional fraction of local radiotherapy (Bragg mode). Two weeks after starting the combination, she developed grade 4 myelosuppression, and all antineoplastic drugs were withheld on February 4, 2026. After supportive care and hematologic recovery, she continued vebreltinib monotherapy (200 mg twice daily) from February 14, 2026.

## Patient update

A restaging CT performed on March 17, 2026 demonstrated a PR per RECIST v1.1 (Figure 3A): the dominant liver lesion decreased from 53 × 34 mm to 47 × 30 mm, and CA19-9 normalized to 30.7 U/mL (a 99.2% reduction) (Figure 3B). The response was maintained during continued vebreltinib monotherapy. At the follow-up on May 26, 2026, CT showed the liver lesion measuring 29 × 46 mm with no new lesions (Figure 3A); CA19-9 remained normal at 18.5 U/mL (Figure 3B). The patient tolerated vebreltinib monotherapy well, with the response maintained until August 2026, when restaging CT revealed disease progression. Restaging CT on August 5, 2026, showed new pulmonary metastases and enlarged retroperitoneal and iliac lymph nodes, consistent with progressive disease. Serum CA19-9 rose to 83.7 U/mL on August 4 and further increased to 161 U/mL on August 26, 2026. On August 7, 2026, surufatinib (200 mg once daily), an anti-angiogenic tyrosine kinase inhibitor, was added to ongoing vebreltinib (200 mg twice daily), and durvalumab (1500 mg) was reintroduced. The patient continues to receive this triple combination therapy at the latest follow-up.

**Figure 3.**
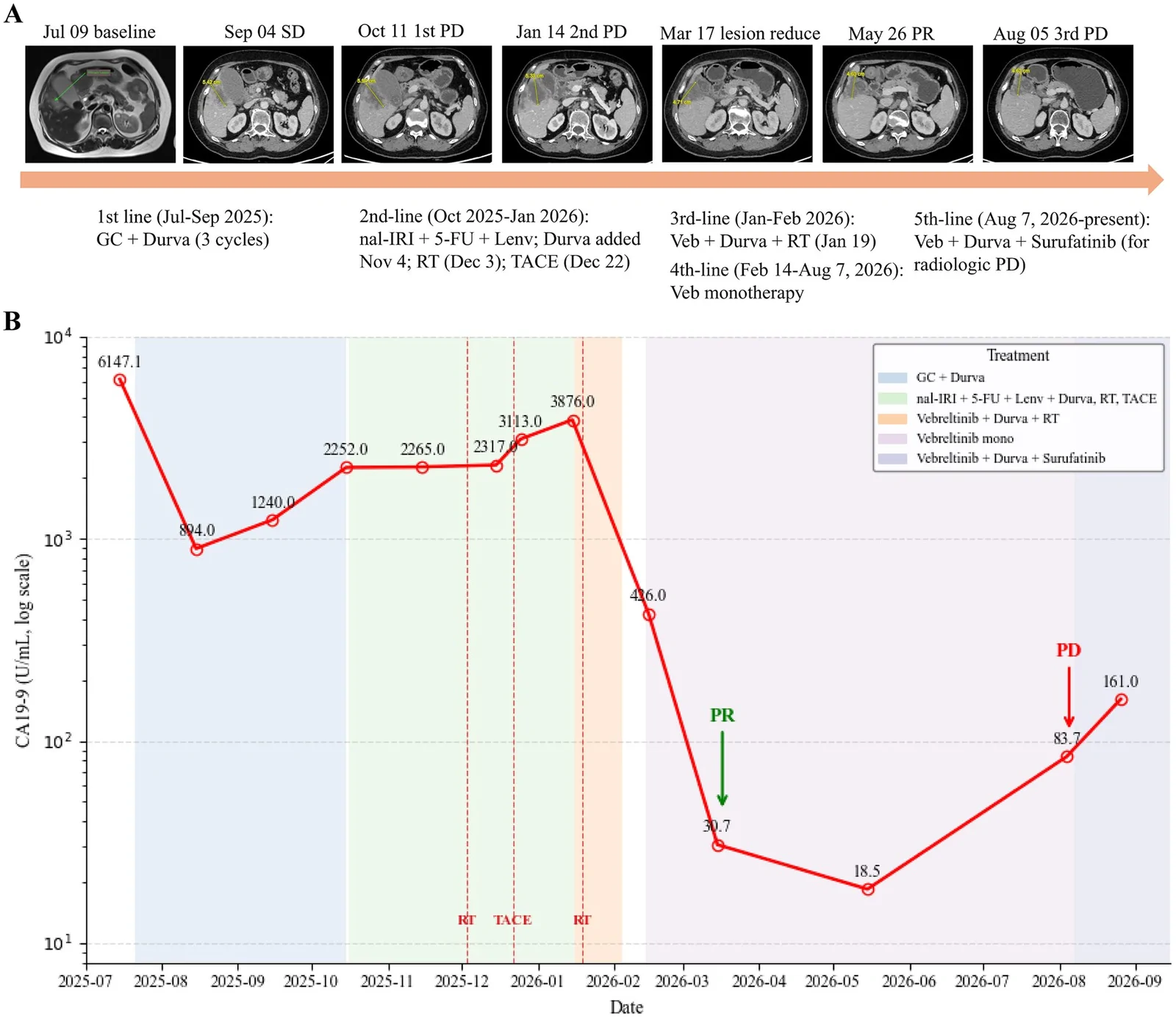
Treatment timeline, imaging response, and dynamics of CA19-9 levels. (A) Axial baseline MRI and serial follow-up axial contrast-enhanced CT images with a corresponding treatment timeline. The baseline MRI (July 09) demonstrates the morphological features of the hepatic lesion without quantitative diameter measurement. On serial CT images, the maximal tumor diameter is marked with calipers for longitudinal size assessment, and radiologic responses (SD, PD, and PR) are labeled above each CT panel. The treatment regimens and lines of therapy are displayed below the imaging panels in chronological order. (B) Serum CA19-9 levels plotted on a logarithmic scale. Background shading denotes distinct treatment phases; dashed lines mark radiotherapy (RT) and transarterial chemoembolization (TACE); arrows indicate PR (March 2026) and PD (August 2026). Abbreviations: 5-FU, fluorouracil; Cis, cisplatin; CT, computed tomography; Durva, durvalumab; Gem, gemcitabine; Lenv, lenvatinib; nal-IRI, liposomal irinotecan; PD, progressive disease; PR, partial response; RT, radiotherapy; SD, stable disease; TACE, transarterial chemoembolization.

## Discussion

ICC is the second most common primary liver cancer, accounting for approximately 15%-20% of all primary liver cancers. It typically presents without early symptoms, grows aggressively, and carries a poor prognosis.^1^ Surgery is the only potentially curative option, but most patients are diagnosed at an advanced stage. Even among those who undergo successful resection, the 5-year overall survival rate remains below 50%.^2^ For patients with unresectable or metastatic disease, standard first-line regimens combine gemcitabine/cisplatin with durvalumab (TOPAZ-1) or pembrolizumab (KEYNOTE-966), with median overall survival benefits of approximately 12-13 months.^3^^,^^4^ For patients who progress and harbor no actionable alterations, fluoropyrimidine plus oxaliplatin or liposomal irinotecan are recommended second-line options.^5^^,^^6^ However, median overall survival remains around 1 year.^7^

For patients with actionable genetic alterations, targeted therapies have provided new opportunities in ICC, including those with *FGFR2* fusions, *IDH1* mutations, and *HER2* amplification. The *MET* receptor tyrosine kinase is another promising target, as *MET* can be activated by amplification, mutation, or fusion.^8^ Although *MET* amplification occurs in only 2%-5% of ICC cases, it is an actionable driver.^9^ Current evidence from case reports and small series indicates that *MET*-targeted therapy can induce durable responses in heavily pretreated patients, with several inhibitors showing activity in *MET*-altered biliary tract cancers.^10–14^ However, these findings are limited by small sample sizes, and larger studies are needed to guide treatment selection.

Available data on vebreltinib, although still limited, have shown promising activity. In the phase II SPARTA trial, vebreltinib achieved an objective response rate (ORR) of 43% in *MET* fusion-positive solid tumors, including a confirmed PR in ICC.^15^ In the KUNPENG trial, vebreltinib demonstrated a 75% response rate, a median time to response of 1.4 months, and a median duration of response of 15.9 months.^16^ Its favorable efficacy may be partly attributed to its pharmacological profile. Vebreltinib is a highly selective type Ib *MET* inhibitor, as opposed to type Ia multikinase inhibitors such as crizotinib, which targets *MET*, *ALK*, and *ROS1*. This greater selectivity may reduce off-target toxicity and enhance antitumor activity.^17^ Although these data were generated in non-small-cell lung cancer and fusion-positive tumors, *MET* exon 14 skipping and *MET* amplification share constitutive *MET* activation, suggesting vebreltinib may be active in *MET*-amplified ICC. Our case provides the first clinical evidence supporting this possibility. However, acquired resistance eventually emerged, highlighting the need for further investigation into resistance mechanisms and combination strategies.

Definitions of high-level *MET* amplification vary across studies, with commonly used thresholds including *MET* GCN of 6 or greater, *MET/CEP7* ratio of 2.0 or greater, or GCN of 10 or greater.^18^ However, with a tissue GCN of 26.79 and a plasma GCN of 9.65, our patient’s amplification level far exceeds these cutoffs. High *MET* GCN correlates with greater oncogene addiction and enhanced sensitivity to *MET* inhibitors.^18^ In the phase II GEOMETRY mono-1 trial, patients with *MET* GCN ≥10 achieved an ORR of 29%, compared with 12% in those with GCN 6-9.^19^ Similarly, in the phase II INSIGHT 2 trial, patients with *MET* GCN ≥10 achieved an ORR of 56.6%.^20^ Our patient’s GCN of 26.79 falls into the highest sensitivity category, explaining the rapid tumor regression: CA19-9 decreased by 99.2%, and the target lesion shrank from 53 × 34 mm to 29 × 46 mm (Figure 3A and B).

Detection of *MET* amplification in both tissue and plasma (GCN 26.79 vs 9.65) supports the utility of liquid biopsy as a complementary tool when tissue samples are limited.^21^ Furthermore, serial monitoring of plasma circulating tumor DNA during vebreltinib therapy could potentially detect emerging resistance mutations, such as *MET* D1228N or Y1230C, earlier than radiographic progression. Although such monitoring was not performed in this case, this approach warrants exploration in future studies.

Our patient also carried a *TP53* exon 5 p. F134L missense mutation. This co-mutation did not appear to blunt the efficacy of vebreltinib, consistent with other reports in biliary tract cancers where *TP53* mutations did not preclude durable responses to *MET* inhibitors.^13^ The low TMB (4.1 mut/Mb) and microsatellite stable status likely explain the limited benefit from prior immunotherapy,^22^ reinforcing that *MET* amplification, rather than immune checkpoint activity, was the dominant driver in this patient.

This case offers several clinical implications. First, it supports that *MET* amplification may confer primary resistance to first-line immunochemotherapy. For patients with high-level *MET* amplification at diagnosis, frontline *MET* inhibitor-based therapy could be explored as a potential alternative, though this requires prospective validation in larger cohorts. Second, the disease course underscores the importance of early comprehensive molecular profiling after first-line failure. Identification of *MET* amplification enabled a targeted therapy, with which the patient had a greater than 99% decline in CA19-9 and a durable PR lasting over 6 months, a magnitude rarely achieved in later-line ICC. Third, our case addresses an unmet need in this rare subtype and proposes vebreltinib as a viable choice after multiple standard therapies have failed.

This case has several limitations. As a single observation, it cannot establish generalizable efficacy. Follow-up was limited to 6 months, during which acquired resistance emerged. Longer follow-up in larger cohorts is needed to characterize resistance mechanisms and evaluate combination strategies. Molecular monitoring for resistance was not performed. Despite these limitations, to our knowledge, this is the first report of vebreltinib demonstrating deep and durable antitumor activity in *MET*-amplified ICC.

## Supplementary Material

oyag409_Supplementary_Data

## Data Availability

All data generated or analyzed during this study are included in this article. Further de-identified data are available from the corresponding author upon reasonable request.

## Glossary

**Gene copy number (GCN):** The number of copies of a particular gene in the genome

**MET amplification:** An increase in MET gene copy number leading to constitutive receptor activation and tumor growth

**Tumor mutational burden (TMB):** The total number of somatic mutations per megabase of the genome

**Microsatellite stable (MSS):** The absence of microsatellite instability, indicating a genomically stable tumor
