# Supplementary material for: Durable response to vebreltinib in heavily pretreated MET-amplified intrahepatic cholangiocarcinoma: a case report
Source: Oncologist. 2026 Oct 13;31(11):oyag409. doi: 10.1093/oncolo/oyag409 (PMC13685772; doi:10.1093/oncolo/oyag409)
Supplement: Supplementary file 1 [file oyag409_supplementary_data.zip › Supplementary Method.docx]

Targeted DNA panel sequencing

FFPE tumor tissues, plasma samples, and matched peripheral blood leukocytes were collected for targeted DNA panel sequencing. FFPE tumor sections were reviewed to ensure adequate tumor content, with a minimum tumor cell fraction of 10% and necrotic tissue less than 70%. Genomic DNA was extracted from FFPE tumor tissues and matched leukocytes using QIAGEN DNA extraction kits, while circulating cell-free DNA was isolated from plasma using the QIAGEN Circulating Nucleic Acid Kit according to the manufacturer’s instructions. Sequencing libraries were prepared after genomic DNA fragmentation or cfDNA purification, followed by end repair, A-tailing, adaptor ligation, purification, and PCR amplification. Target enrichment was performed using a hybridization capture-based 425-gene cancer panel developed by Nanjing Geneseeq Technology Inc. The enriched libraries were sequenced on an Illumina HiSeq platform with paired-end 150-bp reads, with mean sequencing depths of at least 1,000× for FFPE tumor tissue, 5,000× for plasma cfDNA, and 100× for matched leukocyte controls.

Raw sequencing data were converted to FASTQ format using bcl2fastq and subjected to quality control. Clean reads were aligned to the human reference genome hg19/GRCh37 using BWA-MEM, followed by post-alignment processing with Picard and GATK. Single-nucleotide variants and small insertions/deletions were identified using VarScan2 and annotated with ANNOVAR. Copy number variations were analyzed using ADTEx based on normalized sequencing depth across targeted regions, with correction for GC content, capture efficiency, local background variation, and matched leukocyte control signals. Gene amplification was defined as a significant gene-level copy number gain exceeding the validated assay-specific threshold after adjustment for tumor purity or circulating tumor DNA fraction when available, and was required to be supported by concordant coverage elevation across multiple captured exons or probes. Candidate variants were manually reviewed using Integrative Genomics Viewer when necessary.
